# An effective method for preparing histological sections of blow fly intra-puparial stages for minimum PMI estimations

**DOI:** 10.1007/s00414-024-03211-5

**Published:** 2024-04-09

**Authors:** Daniel Martín-Vega, Thomas J. Simonsen, Martin J. R. Hall

**Affiliations:** 1 Departmento de Ciencias de la Vida, Universidad de Alcalá, Alcalá de Henares, Madrid, 28805 Spain; 2 Natural History Museum, Cromwell Road, London, SW7 5BD UK; 3 Natural History Museum Aarhus, Aarhus C, 8000 Denmark

**Keywords:** Forensic entomology, Insect anatomy, Insect development, Metamorphosis, Morphological analysis, Post-mortem interval, Pupal stage

## Abstract

Blow flies (Diptera: Calliphoridae) are generally early colonisers of fresh cadavers, enabling the estimation of a minimum post-mortem interval (_min_PMI) based on an accurate aging of the oldest immature stages associated with a cadaver. In blow flies, the pupal stage and the subsequent development of the adult take place inside a protective case, the puparium, formed from the hardened and darkened cuticle of the third instar larva. Because the puparium is an opaque structure that shows virtually no external changes, qualitative analyses of the internal tissues can be very informative for determining reliable age-specific morphological markers. Those analyses can be performed using either non-invasive but expensive and not widely accessible techniques, or traditional histological methods, which are invasive as they require the serial sectioning of the sample. Histological methods are often readily available for forensic researchers and practitioners; however, the histological study of blow fly intra-puparial stages has traditionally been hampered by the poor paraffin infiltration of tissues due to the abundance of fat bodies, resulting in usually fragmented sections and the subsequent loss of relevant information. We present here an effective method for the preparation of histological sections of blow fly intra-puparial stages, maximising the paraffin infiltration while enabling the production of clean and entire sections that allow for the use of reliable age-specific morphological markers, thus improving the accuracy of _min_PMI estimations when access to more costly techniques is not feasible.

## Introduction

Highly synanthropic and present in most habitats, blow flies (Diptera: Calliphoridae) are generally early colonisers of fresh cadavers and, therefore, ideal forensic indicators [1]. Colonisation here refers to the first batch of eggs laid by the blow fly females arriving to the cadaver, followed by a typical holometabolous insect life cycle, where a larval stage transforms into an anatomically different adult through a non-feeding, quiescent stage, called a pupa [2, 3]. The process of dramatic morphological changes during the pupal stage is known as “complete metamorphosis”, and it implies an extensive remodelling of organs and tissues through processes of histolysis and histogenesis [4, 5]. In cyclorrhaphous flies, which include the Calliphoridae and other Diptera families, the pupal stage and the subsequent development of the adult take place inside a protective case, the puparium, formed from the hardened and contracted cuticle of the third instar larva [3]. This intra-puparial period of the blow fly life cycle comprises more than 50% of the total time required for the immature development [1, 3]; hence, it can be crucial in minimum post-mortem interval (_min_PMI) estimations, as they often rely on an accurate aging of the oldest immature stages of blow flies associated with the cadaver [1].

Aging intra-puparial stages can be particularly challenging, because the puparium is an opaque structure that shows virtually no external changes beyond the first few hours after pupariation, when it darkens due to hormonally induced melanisation [6]. Although different methods for aging intra-puparial stages of blow flies have been developed, those based on qualitative morphological analyses are the most promising for forensic casework [7]. A simple aging method consists in dissecting the puparium to perform an external morphological analysis of the insect inside [8, 9]. However, although this is a low-cost and valuable method, its reliance on colouration characters can limit the accuracy and reliability of the analysis if, for example, some tissues and structures are discoloured during preservation and storage [10]. In this sense, a combination of external characters with internal morphological analyses of histological sections can significantly improve the accuracy of age estimates, as they provide a range of unambiguous qualitative changes in the internal tissues and organs during metamorphosis [4, 5, 8, 11, 12]. Internal morphological analyses can be performed using either traditional histology [8, 11] or novel imaging techniques, like X-ray micro-computed tomography (micro-CT) [2, 4, 5, 12–15]. Unlike traditional histology, micro-CT analyses have the advantage of being non-invasive, providing high-resolution, virtual histological sections of the same specimen in any plane, and enabling the acquisition of accurate volumetric data of internal organs that can be used as a quantitative measure of age in combination with qualitative markers [2, 4, 5, 12, 15]. On the other hand, performing micro-CT scanning and analyses require specific training and expertise, and the access to this technology might not be available to many researchers and practitioners [12]. In contrast, traditional histological methods are invasive—the specimens are actually sectioned in a specific plane—, but the required facilities are more readily available as they are cheaper and routinely used in many medicolegal institutions [16].

There is a variety of protocols and techniques specifically developed for the histological study of different types of insect material using traditional techniques [17]. In the specific case of the intra-puparial stages of cyclorrhaphous flies, however, preparing histological sections is particularly challenging [2, 11]. As noted by Brown et al. [10] and Davies et al. [11], the abundance of fat bodies in both pupal and pharate adult specimens inhibits the paraffin infiltration, resulting in brittle and usually fragmented sections. To facilitate the penetration of paraffin, Davies et al. [11] recommended a bisection of the intra-puparial specimens between the thoracic and abdominal regions, and piercing the insect with a pin, once throughout each tagma. Nevertheless, that type of manipulation might pose the risk of severely damaging age-informative internal organs and tissues, and it may result in heavily fragmented sections anyhow [11]. We present here an effective and refined method for preparing histological sections of blow fly intra-puparial stages, maximising the paraffin infiltration with no need for prior puncturing or bisection of the specimens. This method enables the preparation of clean and entire histological sections that allow for the use of reliable internal morphological markers for aging intra-puparial stages [4, 12, 14, 15], improving the accuracy of _min_PMI estimations.

## Materials and methods

A laboratory colony of the blue-bottle blow fly, *Calliphora vicina* Robineau-Desvoidy was established and maintained at the Natural History Museum, London, following Martín-Vega et al. [4, 5]. Following a standard protocol for blow fly rearing [18], *C. vicina* immatures were cultured at a constant temperature (24 °C ± 0.8 °C) without light, and puparia from different ages were sampled as detailed in previous publications [4, 5, 13, 15]. Sampling times were established using percentages of the total intra-puparial period (IPP), with 0% corresponding to pupariation and 100% to adult emergence [4, 5]. Four puparia were collected at random at each 10, 20, 50 and 100% IPP, as representative time points of the intra-puparial development [3, 5]. The collected puparia were killed and fixed in hot, near boiling water for approximately 30 s [10] without piercing, and then stored in 80% ethanol at 4 °C until histological analysis.

For histological analyses, the puparia were dissected to extract the insect inside and facilitate paraffin infiltration. For dissection, the puparium was placed in a glass cavity block with 80% ethanol and held with forceps. A scalpel incision was made around the first thoracic segment, enabling separation of the anterior end of the puparium, and subsequent gentle extraction of the insect with forceps. A first (and unsatisfactory) trial was conducted following a standard protocol for histological analyses used at the Royal Botanic Gardens, Kew [19]: two intra-puparial specimens from each IPP percentage batch were dehydrated through an ethanol series to 100% ethanol and then cleared through an ethanol–Histoclear® series to 100% Histoclear®, using a Leica® TP1020 tissue processor. The cleared samples were embedded in Paraplast® and sectioned in 10 μm thick sections on a Leica® Reichert-Jung 2040 microtome. The resulting sections were stained using a trichrome stain: Weigert’s haematoxylin, bluish erythrosine, and fast green preceded by phosphomolybdic acid [20, 21]. The above method gave very unsatisfactory results, with very fragmented sections, and similar issues to those noted by Davies et al. [11].

A second (and successful) trial was conducted on the remaining two intra-puparial specimens from each IPP percentage group; this time, butanol was used for dehydration (Kristensen NP, unpublished teaching material): the samples were transferred from 80% ethanol to a 1:1 solution of 70% ethanol/butanol for 2 h, and then transferred to pure butanol for 12 h. Simultaneously, a solution of butanol saturated with Paraplast® pellets was left at 40 °C for 12 h to ensure the melting of the pellets. The samples were then transferred from pure butanol to butanol saturated with Paraplast® for 4 h at room temperature, with one change after 2 h; then transferred to fresh butanol saturated with Paraplast® for 30 min at 40 °C, and then transferred to pure Paraplast® at 60 °C in a vacuum oven at 500 mbar for 3 h, changing to fresh Paraplast® every hour, before a final change to fresh Paraplast® and leaving in the 60 °C vacuum oven overnight. When the samples were removed from the oven, they were first transferred to liquid Paraplast® in a small aluminium tray on a hot plate. When the tray was removed from the hot plate, the Paraplast® rapidly cooled and solidified, thereby embedding the sample in a Paraplast® block. The blocks were sectioned in the sagittal plane, in 10 μm thick sections on a Leica® Reichert-Jung 2040 microtome. The resulting sections were stained using a trichrome stain [20, 21] as described above, and mounted under cover slips in DPX (Dibutylphthalate Polystyrene Xylene) on Polysine® adhesion microscope slides. Photographs were taken using a Leica® DM6000 B microscope and a Leica® M165 stereo microscope.

## Results and discussion

**Fig. 1:**
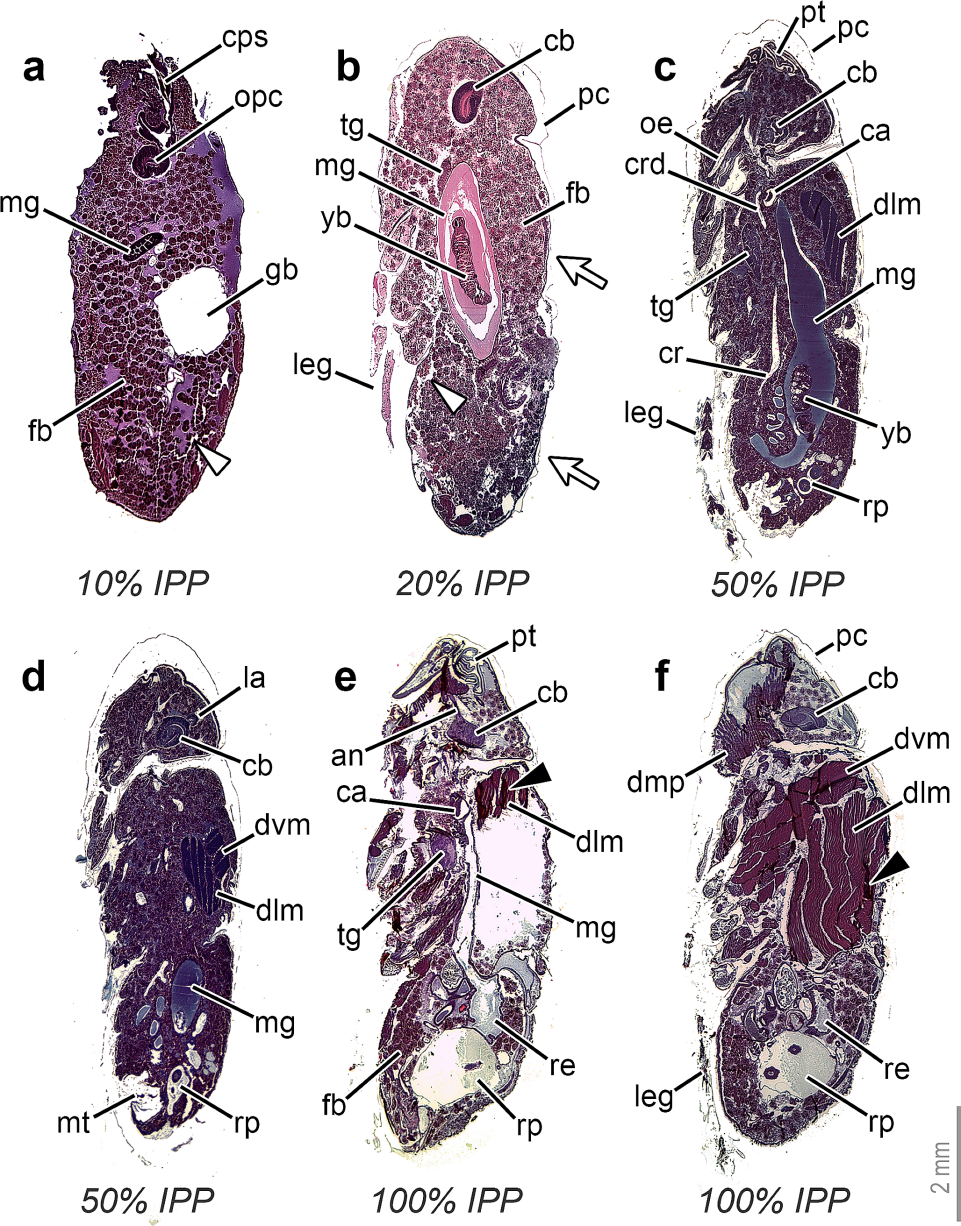
Histological sagittal sections of *Calliphora vicina* at different percentages of time of the total intra-puparial period (IPP). **a** 10% IPP, medial sagittal section. **b** 20% IPP, medial sagittal section. White arrows indicate areas where the pupal cuticle is still attached to the adult epidermis. **c** 50% IPP, medial sagittal section. **d** 50% IPP, lateral sagittal section. **e** 100%, medial sagittal section. **f** 100%, lateral sagittal section. *an* antennal nerve, *ca* cardia or proventriculus, *cb* central brain, *cps* cephalopharyngeal skeleton, *cr* crop, *crd* crop duct, *dlm* dorsal lateral muscles, *dvm* dorsoventral muscles, *fb* fat bodies, *gb* gas bubble, *la* lamina, *leg* leg, *mg* midgut, *mt* male terminalia, *oe* oesophagus, *opc* outer proliferation centre of brain hemisphere, *pc* pupal cuticle, *pt* ptilinum, *re* rectum, *rp* rectal pouch, *tg* thoracic ganglion, *yb* yellow body. White triangles indicate areas where the internal tissues were fragmented during processing. Black triangles indicate areas with wrinkles in muscle fibres

The method presented here resulted in complete and fully informative sagittal sections for the four selected IPP percentages (Fig. 1). Although a few cracks (Fig. 1a and b) and wrinkles (Fig. 1e and f) were observed in some sections, they were usually minimal and did not hinder the observation of age-specific morphological markers [4, 12]. Whereas wrinkles appeared at muscle tissue in 100% IPP specimens, cracks were mainly present in the internal tissues at 10% and 20% IPP, corresponding to the cryptocephalic and phanerocephalic pupal stages [3], respectively. These are the periods of most drastic changes, including the eversion of head and legs at 20% IPP [13]. Consequently, the specimens typically show a profusion of fat bodies (Fig. 1a and b) and extensive histolysis and histogenesis [5] that make the appropriate paraffin infiltration of tissues difficult. Nevertheless, the histological sections obtained unequivocally showed the morphological landmarks of these development intervals [4]. At 10% IPP, the partially extruded cephalopharyngeal skeleton [4], the crescent-shaped outer proliferation centre of the brain hemispheres [15] and the large, characteristic gas bubble within the internal tissues of the abdominal region [4, 13] were all observed (Fig. 1a). At 20% IPP, the adult midgut showed a closed-sack shape in sagittal view, occupying the central body region and containing the apoptotic larval midgut or “yellow body”, whereas the thoracic ganglion was located at the most anterior part of the thorax (Fig. 1b). Both characters are diagnostic markers for that development interval [4]. Moreover, the separation of the pupal cuticle from the adult epidermal cells was observed only in some areas (Fig. 1b), indicating that the pupal-adult apolysis was ongoing but not complete [5]. Determining if an apolysis event is complete is crucial for an accurate delimitation of the prepupal, pupal and pharate adult stages [3], but this information could be easily lost if the histological sections are not properly prepared and processed.

**Fig. 2:**
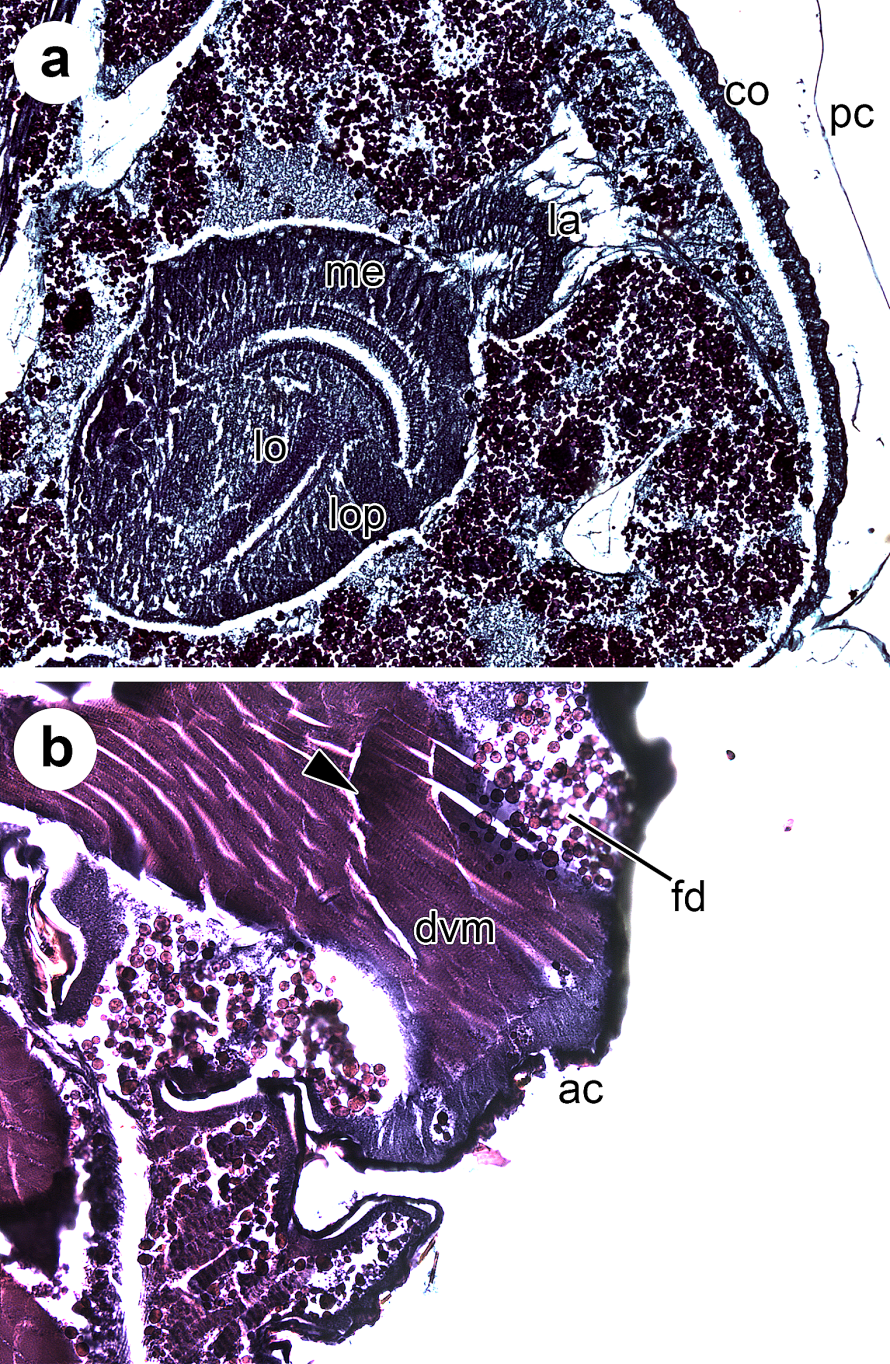
Histological sagittal sections of *Calliphora vicina* at different percentages of time of the total intra-puparial period (IPP). **a** 50% IPP, lateral sagittal section of the head, showing the “horseshoe-shaped” lamina of the brain. **b** 100% IPP, lateral sagittal section of the thorax, showing the attachment of the indirect flight muscles to the adult cuticle. Black triangle indicates an area with wrinkles in the muscle fibres. *ac* adult cuticle, *co* cornea, *dvm* dorsoventral muscles, *fd* fatty droplets, *la* lamina, *lo* lobula, *lop* lobula plate, *me* medulla, *pc* pupal cuticle

The pharate adult stage, which begins once the pupal-adult apolysis is complete [3], is a period of less profound morphological changes than the pupal stage [5]. Nonetheless, it is still possible to determine the IPP percentage during this stage on the basis of age-diagnostic morphological characters [4, 12, 14, 15], all of them clearly observable in the present histological sections (Fig. 1c and f). During the earlier development of the pharate adult, the shape of the adult midgut and the position of the crop are particularly age-informative [4, 5]. As the crop develops, it lengthens and extends from the anterior part of the thorax, reaching its final position in the anterior region of the abdomen at 50% IPP [4]. The present histological sections showed this age-diagnostic character unambiguously (Fig. 1c). The pre-helicoidal region of the adult midgut, which shows a “long-necked bottle” shape in sagittal view at 40% IPP [4], starts to widen uniformly along its length and acquires a more tubular shape at 50% IPP, as observed in the present sections (Fig. 1c). Furthermore, at 50% IPP the indirect flight muscle fibres are easily discernible (Fig. 1c and d) yet not fully formed nor attached to the adult cuticle [4]. In the brain, the lamina, one of the neuropils that form the optic lobe, shows a characteristic “horseshoe” shape at 40% and 50% IPP (Figs. 1d and 2a), before unfolding and extending below newly formed retinular cells at 60% IPP [4, 15].

Finally, at 90–100% IPP, the internal organs and tissues are fully developed and the pharate adult is ready to emerge from the puparium [5]. Particularly noteworthy is the development of the head and thoracic muscles (Fig. 1e and f), which only attach to the adult cuticle (Fig. 2b) at the end of the intra-puparial development [4, 5]. At 100% IPP, the histological sections often showed wrinkles in the muscle fibres (Fig. 1e and f), even in the median plane, where, at that IPP percentage, there is typically no tissue between the two sets of dorsal longitudinal indirect flight muscles in some areas of the thorax [4]. Anyhow, those wrinkles did not hamper the observation of the muscle architecture (Fig. 1e and f), as fully formed indirect flight muscles that occupy the majority of the thoracic region is a diagnostic character for the last IPP percentages [4, 11, 12, 14]. During intra-puparial development, the muscle fibres are mostly surrounded by haemocytes and fatty droplets [11, 22], which can be an obstacle for the proper infiltration of paraffin (Fig. 2b). On the other hand, the present sections showed a narrow tube-shaped midgut in the thoracic region at 100% IPP (Fig. 1e), in contrast to the appearance of that organ at 50% IPP (Fig. 1c). As the indirect flight muscles grow and fill most of the thorax during intra-puparial development, the pre-helicoidal region of the midgut becomes narrower, being a clear indicator of the intra-puparial age [4, 5, 12]. Whereas the volume of the pre-helicoidal region of the midgut decreases, the lumen of the rectum and rectal pouch increases and at 90–100% IPP is typically filled with meconium [4, 5], which was clearly observable in the present histological sections (Fig. 1e and f).

Brown et al. [10] and Davies et al. [11] recommended a physical fixation of blow fly intra-puparial stages for histological analysis using near boiling water, instead of using chemical fixatives traditionally used in insect histology, like formaldehyde, Kahle’s, Bouin’s or Carnoy’s solutions [8, 17, 20, 21]. Hot water fixation has clear advantages in forensic casework: it is a readily available method and the accepted standard for the killing and fixation of blow fly larvae to enable subsequent analyses [1]. Previous studies using micro-CT-based virtual histology have shown that it is certainly an effective method for the fixation of the internal tissues of intra-puparial stages of cyclorrhaphous flies [4, 5, 12–15, 23], and the present study corroborates that it is also a valid fixation method for preparing traditional histological sections. On the other hand, Brown et al. [10] and Davies et al. [11] recommended piercing with a pin throughout the insect to enable the penetration of the hot water [10] and facilitate the paraffin infiltration of the internal tissues [11]. In addition, Davies et al. [11] recommended the bisection of the intra-puparial form, once extracted from the puparium, to further enhance the paraffin infiltration. We disagree with those recommendations, as piercing and bisection damage the internal tissues and may result in a significant loss of information. Puncturing the puparium (but not the insect inside) after hot water fixation may be useful to enhance the penetration of contrast solutions for micro-CT scanning [4, 5, 12–14, 23], but, for traditional histological analysis, it is preferable to dissect the puparium and extract the insect inside, which can then be taken through the dehydration, clearing and embedding processes with no need for piercing or bisection. Extracting the entire insect from the puparium also has the advantage of enabling an external morphological analysis [8, 9] prior to the internal histological analysis, thereby increasing the accuracy of the age estimation by combining both methods [8, 12]. Special caution should be taken with recently pupariated (i.e., not fully darkened) specimens, because during the first 5–7.5% IPP some parts of the insect body are still attached to the puparial wall (i.e., the larval-pupal apolysis is not complete) [3, 5]; hence, the extraction might be particularly challenging. However, from a forensic perspective this is not an issue, as the stage of insect development will be evident, hence the age and _min_PMI when temperature is taken into account.

To avoid the piercing and bisection of the intra-puparial specimens, we strongly recommend the use of butanol and a vacuum, as it greatly enhances the proper paraffin infiltration of tissues in arthropod samples [17, 20, 24–27]. Butanol is a solvent of paraffin and its advantages over other dehydrating agents like absolute ethanol or xylol have long been highlighted, as it prevents the hardening and shrinking of tissues [17, 24, 25, 27]. It has proven to be particularly useful for the serial sectioning of arthropods with a weakly chitinized cuticle [25, 27], which is the case of both the pupal and pharate adult cuticles [5]. Regarding the use of a vacuum oven, it facilitates the paraffin infiltration of tissues by providing negative atmospheric pressure conditions, thus accelerating the complete removal of solvents and air bubbles [26, 27]. Most modern tissue processors are equipped with a vacuum pump connection [27], so access to this facility should be readily available. Although the procedure described here is more time-consuming than other standard procedures [11], it enables the preparation of sections of the entire specimen (Fig. 1), minimising the risk of damaging informative tissues and structures. In order to optimise time and effort, we recommend sectioning the samples in the sagittal plane, as it enables the visualisation of most age-diagnostic morphological markers, not only for *C. vicina* [4, 5, 12, 15], but also for other forensically relevant Calliphoridae species and other families of cyclorrhaphous flies [4, 14, 23]. Regarding the staining method, the trichrome stain [20, 21] used here has shown to be very effective, but other widely used staining techniques, like haematoxylin-eosin [8, 11], may also provide good results. In conclusion, the method presented here should be easily applicable in most forensic institutions with access to histology facilities, providing an alternative for improving the accuracy of _min_PMI estimations based on intra-puparial samples when access to more expensive techniques is not feasible.

## Data Availability

The datasets generated during the current study are available from the corresponding author upon reasonable request. Not applicable.
